# CCN6-mediated MMP-9 activation enhances metastatic potential of human chondrosarcoma

**DOI:** 10.1038/s41419-018-1008-9

**Published:** 2018-09-20

**Authors:** Huey-En Tzeng, Chih-Hsin Tang, Sz-Hua Wu, Hsien-Te Chen, Yi-Chin Fong, Yung-Chang Lu, Wei-Cheng Chen, Hsien-Da Huang, Chih-Yang Lin, Shih-Wei Wang

**Affiliations:** 1 Taipei Cancer Center, Taipei Medical University, Taipei, 110 Taiwan; 2 Graduate Institute of Cancer Biology and Drug Discovery, College of Medical Science and Technology, Taipei Medical University, Taipei, 110 Taiwan; 3 Division of Hematology/Oncology, Department of Medicine, Taipei Medical University-Shuang Ho Hospital, New Taipei City, 235 Taiwan; 4 Chinese Medicine Research Center, China Medical University, Taichung, 404 Taiwan; 5 Department of Pharmacology, School of Medicine, China Medical University, Taichung, 404 Taiwan; 6 Department of Biotechnology, College of Health Science, Asia University, Taichung, 404 Taiwan; 7 School of Pharmacy, China Medical University, Taichung, 404 Taiwan; 8 School of Chinese Medicine, China Medical University, Taichung, 404 Taiwan; 9 Department of Orthopedic Surgery, China Medical University Hospital, Taichung, 404 Taiwan; 10 Department of Sports Medicine, College of Health Care, China Medical University, Taichung, 404 Taiwan; 11 Department of Orthopaedic Surgery, China Medical University Beigang Hospital, Yun-Lin County, 651 Taiwan; 12 Department of Medicine, Mackay Medical College, New Taipei City, 252 Taiwan; 13 Department of Orthopaedics, MacKay Memorial Hospital, Taipei, 104 Taiwan; 14 Degree Program of Biomedical Science and Engineering, National Chiao Tung University, Hsinchu, 300 Taiwan; 15 Institute of Bioinformatics and Systems Biology, National Chiao Tung University, Hsinchu, 300 Taiwan; 16 Department of Biological Science and Technology, National Chiao Tung University, Hsinchu, 300 Taiwan; 17 Graduate Institute of Natural Products, College of Pharmacy, Kaohsiung Medical University, Kaohsiung, 807 Taiwan

## Abstract

Chondrosarcomas are primary malignant bone tumors that have a poor prognosis. WNT1-inducible signaling pathway protein-3 (WISP-3, also termed CCN6) belongs to the CCN family of proteins and is implicated in the regulation of various cellular functions, such as cell proliferation, differentiation, and migration. It is unknown as to whether CCN6 affects human chondrosarcoma metastasis. We show how CCN6 promotes chondrosarcoma cell migration and invasion via matrix metallopeptidase-9 (MMP)-9 expression. These effects were abolished by pretreatment of chondrosarcoma cells with PI3K, Akt, mTOR, and NF-κB inhibitors or short interfering (si)RNAs. Our investigations indicate that CCN6 facilitates metastasis through the PI3K/Akt/mTOR/NF-κB signaling pathway. CCN6 and MMP-9 expression was markedly increased in the highly migratory JJ012(S10) cell line compared with the primordial cell line (JJ012) in both in vitro and in vivo experiments. CCN6 knockdown suppressed MMP-9 production in JJ012(S10) cells and attenuated cell migration and invasion ability. Importantly, CCN6 knockdown profoundly inhibited chondrosarcoma cell metastasis to lung. Our findings reveal an important mechanism underlying CCN6-induced metastasis and they highlight the clinical significance between CCN6 and MMP-9 in regard to human chondrosarcoma. CCN6 appears to be a promising therapeutic target in chondrosarcoma metastasis.

## Introduction

Chondrosarcomas are common primary malignant bone tumors that are difficult to diagnose and treat^1^. At diagnosis, patients are mostly aged between 30 and 60 years, with a peak between 40 and 50 years. The male:female ratio for chondrosarcoma is ~2:1^1,2^. Chondrosarcomas most frequently involve the scapula, sternum, ribs, and pelvic bones^3^ and their prognosis is poor, as they do not respond well to conventional treatments such as chemotherapy or radiotherapy^4^. Surgical resection is the cornerstone of treatment^5^. The lack of an effective adjuvant therapy for chondrosarcomas highlights the importance of developing novel treatments.

Mortality in cancer patients is mainly due to metastatic spread of cancer cells to distant organs^6^. Extracellular matrix (ECM) surrounding malignant tumor cells has been implicated in almost all stages of the metastatic process^7^. As soon as tumor cells are able to penetrate their surrounding tissue, they are able to pass through the basement membrane and ECM, then penetrate the lymphatic or vascular circulation^8^. Importantly, matrix metalloproteinases (MMPs), also known as matrixins, are calcium-dependent zinc-containing endopeptidases involved in the degradation of the ECM basement proteins in the tumor microenvironment^9^. MMPs also play key roles in vascularization and cell migration^10^. Around 24 types of MMP genes and 23 MMP proteins have been identified to date; all have diverse physiological and pathological functions^11^. Expression levels of MMP-1, MMP-2, MMP-3, MMP-7, MMP-8, MMP-9, and MMP-13 are high in human chondrosarcoma cells^12^.

Cell proliferation, differentiation, adhesion, migration, and invasion are promoted by the CCN family, which contains cysteine-rich 61 (Cyr61, also termed CCN1), connective tissue growth factor (CTGF, also termed CCN2), and nephroblastoma overexpressed (NOV)/CCN3, as well as WISP-1/Elm1 (CCN4), WISP-2/rCop1 (CCN5), and WISP-3 (CCN6)^13,14^. Notably, the CCN (Cyr61, CTGF, and NOV) membrane proteins are essential in tumorigenesis and metastasis^15^. The CNN family also plays regulatory roles in angiogenesis and tumorigenesis^16^. Our previous work indicates that CCN6 regulates metastasis in chondrosarcoma, enhancing chondrosarcoma cell migration by increasing levels of ICAM-1 expression^17^. In this current study, we explored the role of CCN6 in metastasis and upregulation of MMP-9 in human chondrosarcoma cells. We found evidence for the involvement of the phosphatidylinositol 3’-kinase (PI3K), Akt, mTOR, and NF-κB signaling pathways.

## Results

### CCN6-enhanced chondrosarcoma cell migration and invasion involves MMP-9 upregulation

**Fig. 1:**
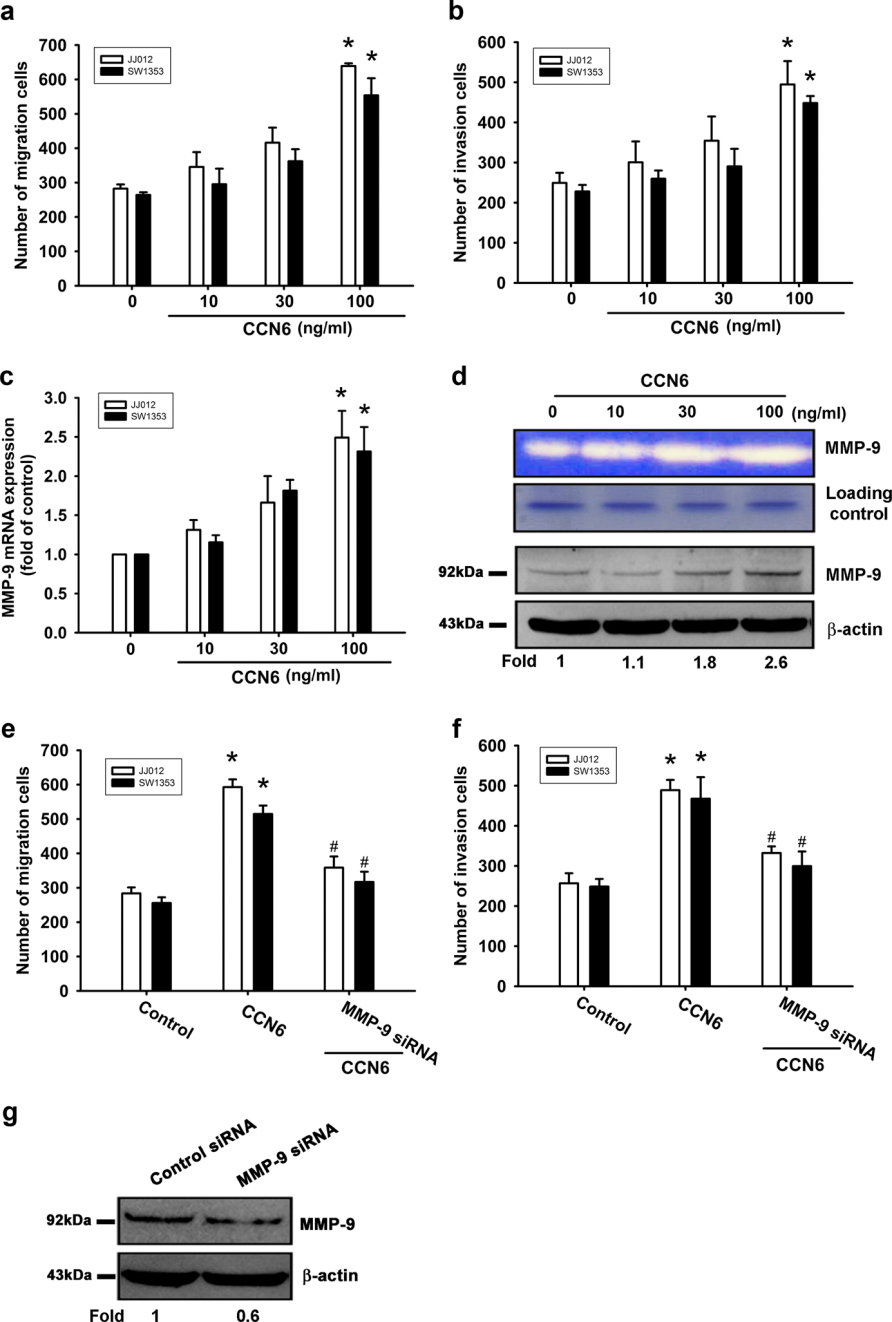
CCN6 increased chondrosarcoma cell migration and invasion, and enhanced cellular MMP-9 expression. **a**, **b** Cells were incubated with CCN6 (10–100 ng/mL), and a Transwell assay determined in vitro migratory and invasion activity after 24 h. **c**, **d** Cells were incubated with CCN6 (10–100 ng/mL) for 24 h, then MMP-9 mRNA and protein expression was examined by RT-qPCR, zymography, and Western blot. **e**, **f** Cells were transfected with MMP-9 siRNA for 16 h then stimulated with CCN6 (100 ng/mL); migration and invasion potential was measured with the Transwell assay. **g** MMP-9 protein levels were measured by Western blot. Quantitative results are expressed as the mean ± SEM. *p < 0.05 as compared with the control group; #*p* < 0.05 as compared with the CCN6-treated group

Our experimental data have shown that CCN6 enhances the wound-healing migration of chondrosarcoma cells by increasing ICAM-1 expression^17^. To confirm these findings, this study used two human chondrosarcoma cell lines (JJ012 and SW1353). Using the Transwell assay, we found that CCN6 dose-dependently stimulated the migratory and invasion activity of human chondrosarcoma cells (Fig. 1a, b). MMP-1, -2, -3, -9 and -13 were expressed in human chondrosarcoma cells^18^. We hypothesized that any of these MMPs might be involved in CCN6-directed chondrosarcoma migration and invasion activity. Stimulation of JJ012 cells with CCN6 significantly induced MMP-9 mRNA expression but not that of other MMPs (see Supplementary Figure S1). Notably, the CCN6-induced increases in MMP-9 mRNA, and protein expression as well as enzyme activity were dose-dependent (Fig. 1c, d). Transfecting cells with MMP-9 siRNA markedly inhibited MMP-9 expression, CCN6-induced cell migration and invasion activity (Fig. 1e–g), which implies that CCN6-induced migration and invasion activity occurs via activation of MMP-9 expression.

### The PI3K/Akt signaling pathway plays a role in CCN6-induced cell migration, invasion, and MMP-9 mRNA expression

**Fig. 2:**
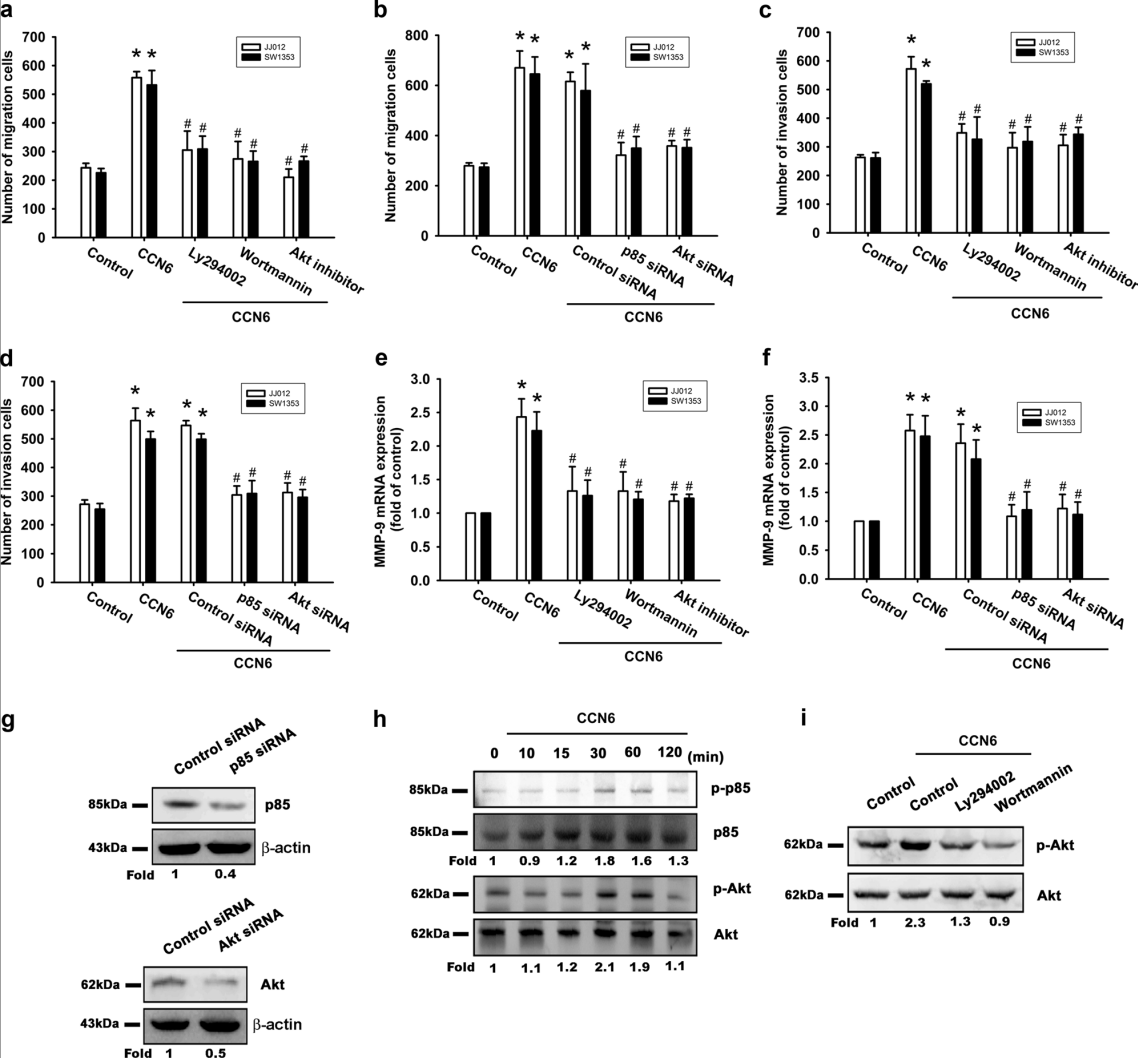
**The PI3K/Akt pathway is involved in CCN6-induced cell migration, invasion, and MMP-9 upregulation in human chondrosarcoma cells.** Cells were pretreated for 30 min with Ly294002 (10 μM), wortmannin (5 μM), and an Akt inhibitor (10 μM), or transfected with siRNA directed against p85 or Akt for 16 h, then stimulated with CCN6. **a**–**d** The Transwell assay examined cell migration and invasion. **e**, **f** MMP-9 mRNA expression was examined by RT-qPCR. **g** p85 and Akt protein levels were measured with Western blot. **h** JJ012 cells were incubated with CCN6 for the indicated time intervals, and p-p85 and p-Akt was examined by Western blotting. **i** JJ012 cells were pretreated with Ly294002 and wortmannin for 30 min, then stimulated with CCN6 for 15 min, and Akt phosphorylation was examined. Quantitative results are expressed as the mean ± SEM. *p < 0.05 as compared with the control group; #*p* < 0.05 as compared with the CCN6-treated group

The PI3K/Akt signaling pathway is commonly implicated in the metastasis of different tumor cells^19,20^. Pretreating chondrosarcoma cells with PI3K inhibitors (Ly294002, wortmannin) or siRNAs abolished CCN6-mediated cell migration, invasion, and MMP-9 mRNA expression (Fig. 2a–f), while transfection of cells with p85 or Akt siRNAs inhibited p85 and Akt expression (Fig. 2g). The PI3K-dependent signaling pathway enzymatically activates Akt residue phosphorylation^21^. When we investigated p85 and Akt phosphorylation in response to CCN6 treatment, we identified a significant, time-dependent induction of p85 and Akt phosphorylation (Fig. 2h). Moreover, CCN6-induced Akt phosphorylation was inhibited when cells were pretreated with p85 inhibitors (Fig. 2i). It appears that CCN6 acts via the PI3K/Akt-dependent signaling pathway to enhance MMP-9 mRNA expression, cell migration, and invasion of human chondrosarcoma cells.

### CCN6 promotes MMP-9 expression and cell motility through the mTOR pathway

**Fig. 3:**
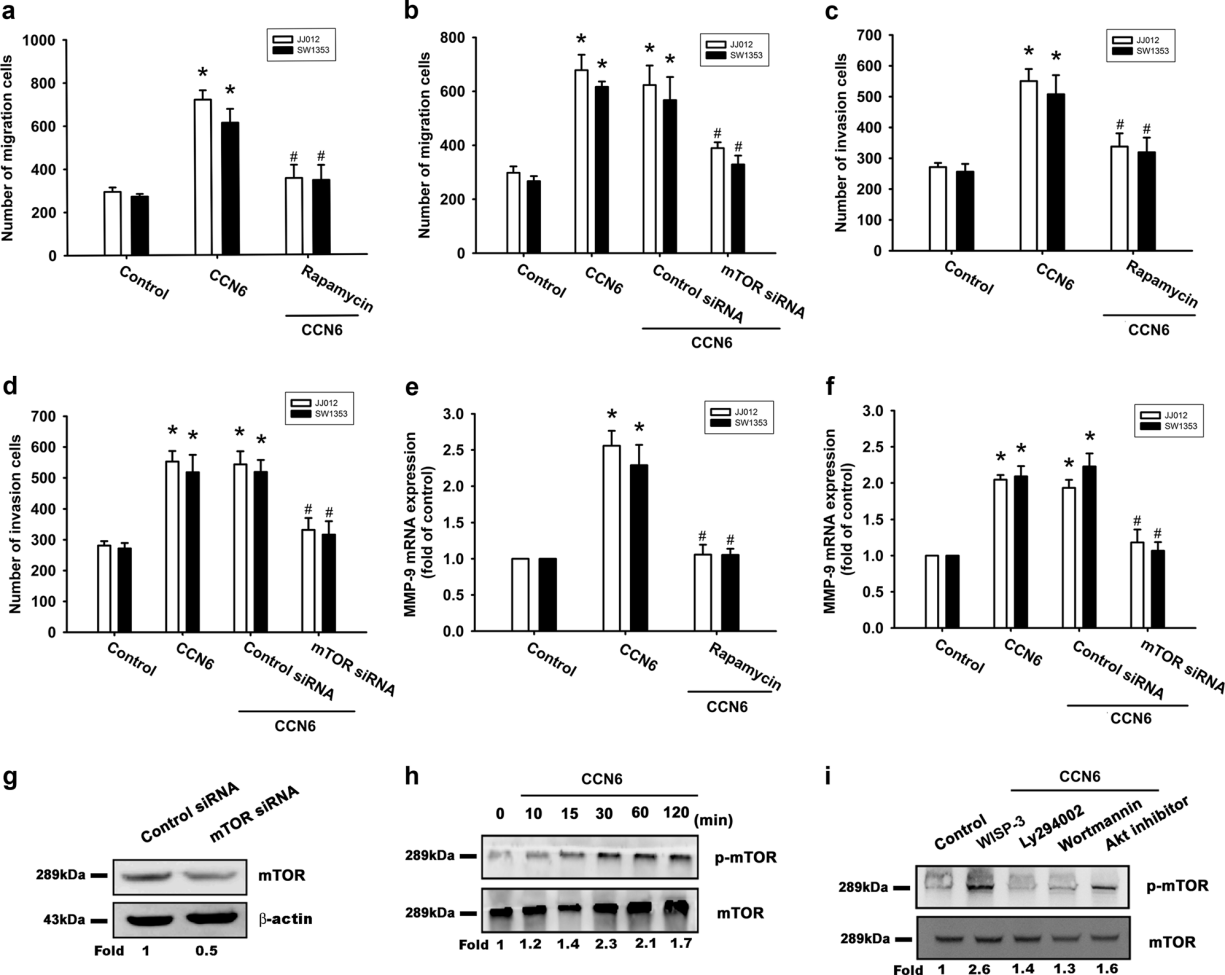
The mTOR pathway is involved in CCN6-induced cell migration, invasion, and increased MMP-9 expression . Cells were pretreated for 30 min with rapamycin (10 μM), or transfected with siRNA directed against mTOR for 16 h, followed by stimulation with CCN6. **a**–**d** Cell migration and invasion were examined using the Transwell assay. **e**, **f** MMP-9 mRNA expression was examined by RT-qPCR. **g** mTOR protein levels were measured using Western blotting. **h** JJ012 cells were incubated with CCN6 for the indicated time intervals, and p-mTOR was examined by Western blotting. **i** JJ012 cells were pretreated with Ly294002, wortmannin, or an Akt inhibitor for 30 min, then stimulated with CCN6 for 15 min, and mTOR phosphorylation was examined. Quantitative results are expressed as the mean ± SEM. *p < 0.05 as compared with the control group; #*p* < 0.05 as compared with the CCN6-treated group

Our analysis of the role of mTOR in CCN6-enhanced cell motility and MMP-9 expression revealed that an mTOR inhibitor (rapamycin) and an mTOR siRNA effectively abolished CCN6-induced cell migration, invasion, and MMP-9 mRNA expression (Fig. 3a–f). Moreover, transfection of cells with an mTOR siRNA inhibited mTOR expression (Fig. 3g). We also found that mTOR phosphorylation in chondrosarcoma cells was increased after CCN6 treatment (Fig. 3h), while incubation of cells with PI3K or Akt inhibitors antagonized CCN6-induced mTOR phosphorylation (Fig. 3i). Thus, the PI3K/Akt/mTOR signaling pathway must be activated for CCN6-induced enhancement of cell migration, invasion, and MMP-9 production in human chondrosarcoma cells.

### NF-κB is involved in CCN6-enhanced cell migration, invasion, and MMP-9 mRNA expression

**Fig. 4:**
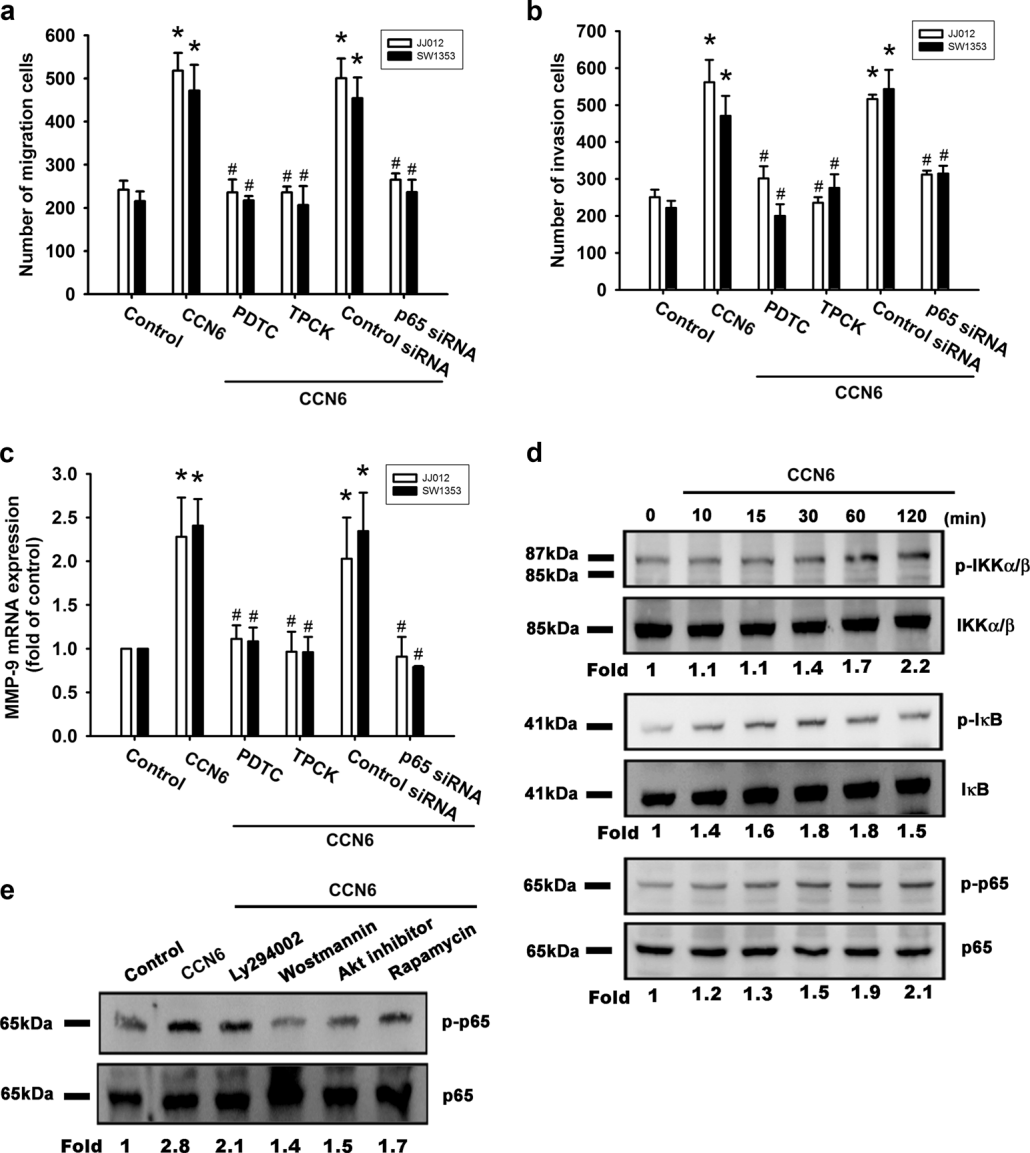
CCN6 induces cell migration, invasion, and MMP-9 upregulation via NF-κB. **a** Cells were pretreated for 30 min with PDTC (10 μM) and TPCK (3 μM) or transfected with p65 siRNA for 16 h, followed by stimulation with CCN6. **a**, **b** Cell migration and invasion were examined using the Transwell assay. (**c**) MMP-9 mRNA expression was examined by RT-qPCR. **d** JJ012 cells were incubated with CCN6 for the indicated time intervals, and p-IKKα/β, p-IκB, and p-p65 levels were examined by Western blotting. **e** JJ012 cells were pretreated with Ly294002, wortmannin, an Akt inhibitor, or rapamycin for 30 min, then stimulated with CCN6 for 60 min, and p65 phosphorylation was examined. Quantitative results are expressed as the mean ± SEM. *p < 0.05 as compared with the control group; #*p* < 0.05 as compared with the CCN6-treated group

**Fig. 5:**
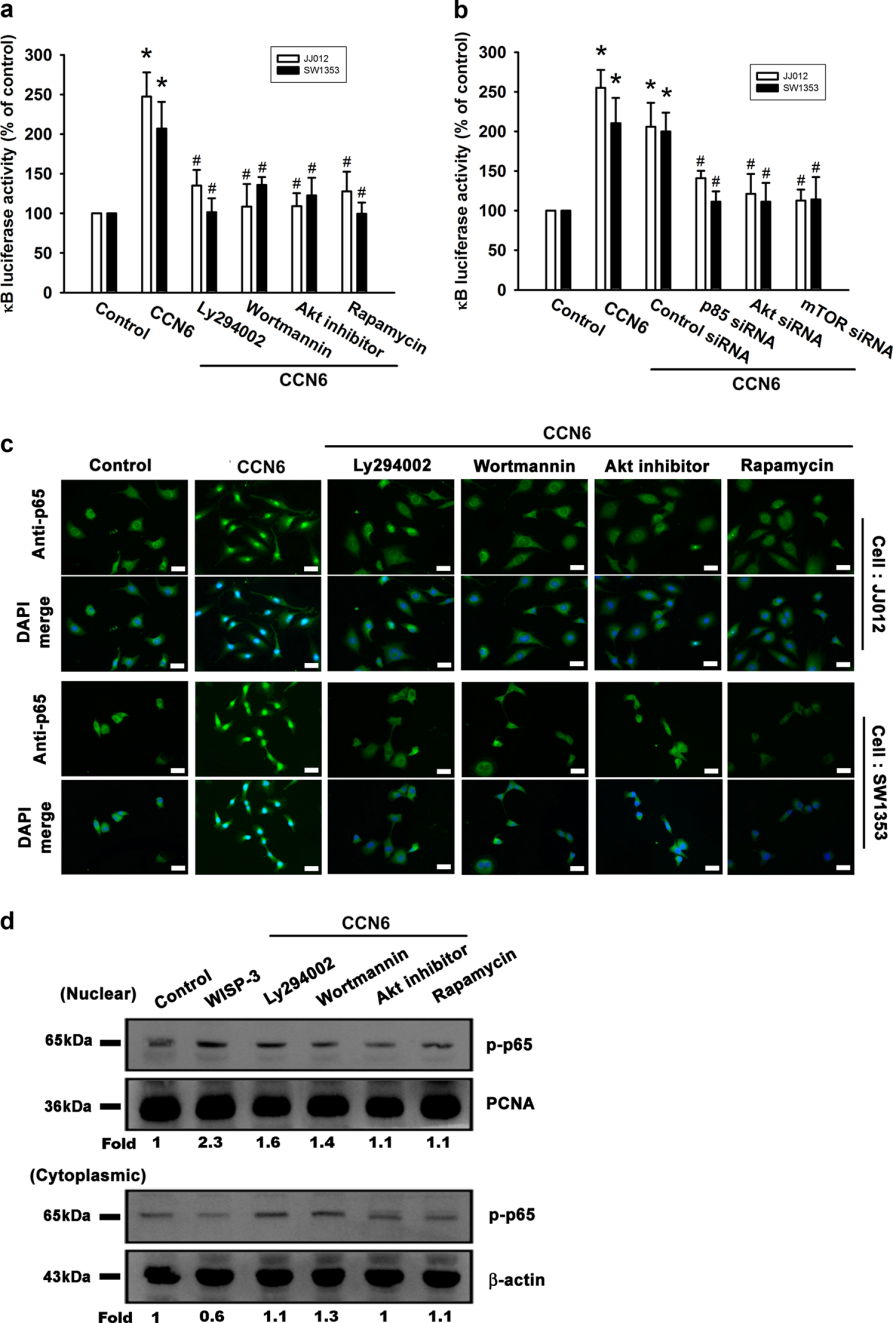
The PI3K/Akt/mTOR pathway is involved in CCN6-mediated NF-κB activation. **a**, **b** Cells were pretreated with Ly294002, wortmannin, an Akt inhibitor, or rapamycin for 30 min, or co-transfected with siRNAs directed against p85 or Akt, or with mTOR and the NF-κB plasmid for 16 h before exposure to CCN6 and subsequent measurement of NF-κB luciferase activity. **c** Cells were pretreated with Ly294002, wortmannin, an Akt inhibitor, or rapamycin for 30 min then stimulated with CCN6 for 60 min, after which the p65 immunofluorescence staining was examined. Size bar = 20 μm. **d** Phosphorylation of p65 expression in the nucleus and cytoplasm was determined by Western blot. Quantitative results are expressed as the mean ± SEM. *p < 0.05 as compared with the control group; #*p* < 0.05 as compared with the CCN6-treated group

Nuclear factor-κB (NF-κB) activation is involved in the migration of human chondrosarcoma cells^22^. We examined whether NF-κB activation plays a role in CCN6-induced activation of the signaling transduction pathway and subsequent chondrosarcoma cell migration, invasion, and enhanced MMP-9 expression. A potent NF-κB inhibitor (PDTC) and a potent IκB protease inhibitor (TPCK) inhibited CCN6-induced enhancement of cell migration, invasion, and increases in MMP-9 mRNA expression (Fig. 4a–c). Transfection with p65 siRNA markedly inhibited CCN6-induced cell migration, invasion, and MMP-9 mRNA expression (Fig. 4a–c). Stimulating chondrosarcoma cells with CCN6 time-dependently promoted IKKα/β, IkBα, and p65 phosphorylation (Fig. 4d). CCN6-induced p65 phosphorylation was significantly reduced when we pretreated the cells with p85, Akt, or mTOR inhibitors (Fig. 4e). We transiently infected chondrosarcoma cells with κB luciferase in order to assess NF-κB activation after CCN6 treatment. Treating chondrosarcoma cells for 24 h with CCN6 increased κB luciferase activity (Fig. 5a); this enhanced activity was blocked when cells were pretreated with PI3K and Akt inhibitors, or rapamycin (Fig. 5a). Co-transfecting cells with p85, Akt, or mTOR siRNAs with κB luciferase plasmid abolished the CCN6-induced increase in κB luciferase activity (Fig. 5b). CCN6-induced accumulation of p65 into the nucleus was also reduced when cells were pretreated with Ly294002, wortmannin, an Akt inhibitor, or rapamycin (Fig. 5c, d). CCN6 appears to depend upon activation of the PI3K, Akt, and mTOR signaling pathways to induce NF-κB activation in human chondrosarcoma cells.

### **Knockdown of CCN6 decreases chondrosarcoma cell migration and invasion, and suppresses MMP-9 expression inin vitroandin vivoanalyses**

**Fig. 6:**
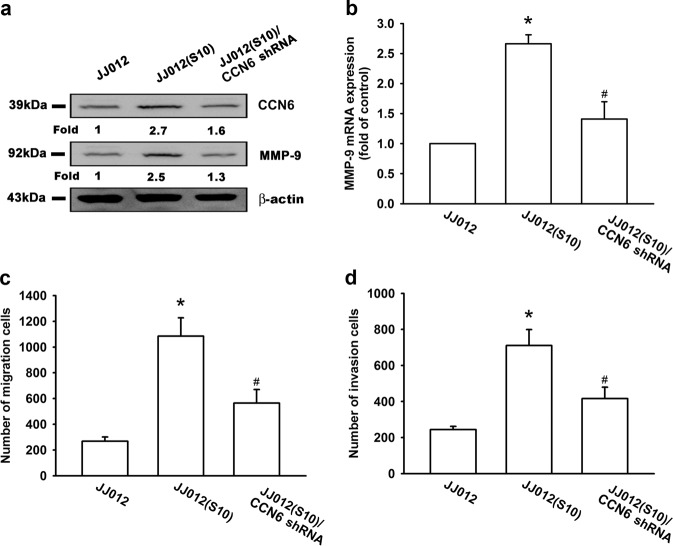
Knockdown of CCN6 decreases cell migration, invasion, and reduces MMP-9 expression in human chondrosarcoma cells. **a**, **b** CCN6 and MMP-9 expression in indicated cells were examined by Western blotting and RT-qPCR. **c**, **d** Migration and invasion activity of chondrosarcoma cells was measured with the Transwell assay. Quantitative results are expressed as the mean ± SEM. *p < 0.05 as compared with the control group; #*p* < 0.05 as compared with the CCN6-treated group

In our previous study, the Transwell assay revealed highly migratory JJ012(S10) cells and we found that CCN6 enhances tumor metastasis in human chondrosarcoma cells^17,23^. JJ012(S10) cells displayed higher CCN6 and MMP-9 expression as compared with JJ012(S0) cells, while CCN6 knockdown effectively reduced CCN6 and MMP-9 expression (Fig. 6a, b). An analysis of JJ012 cells that stably express CCN6 shRNA (JJ012/CCN6 shRNA) revealed that CCN6 knockdown significantly decreased JJ012 cell migration and invasion (Fig. 6c, d).

**Fig. 7:**
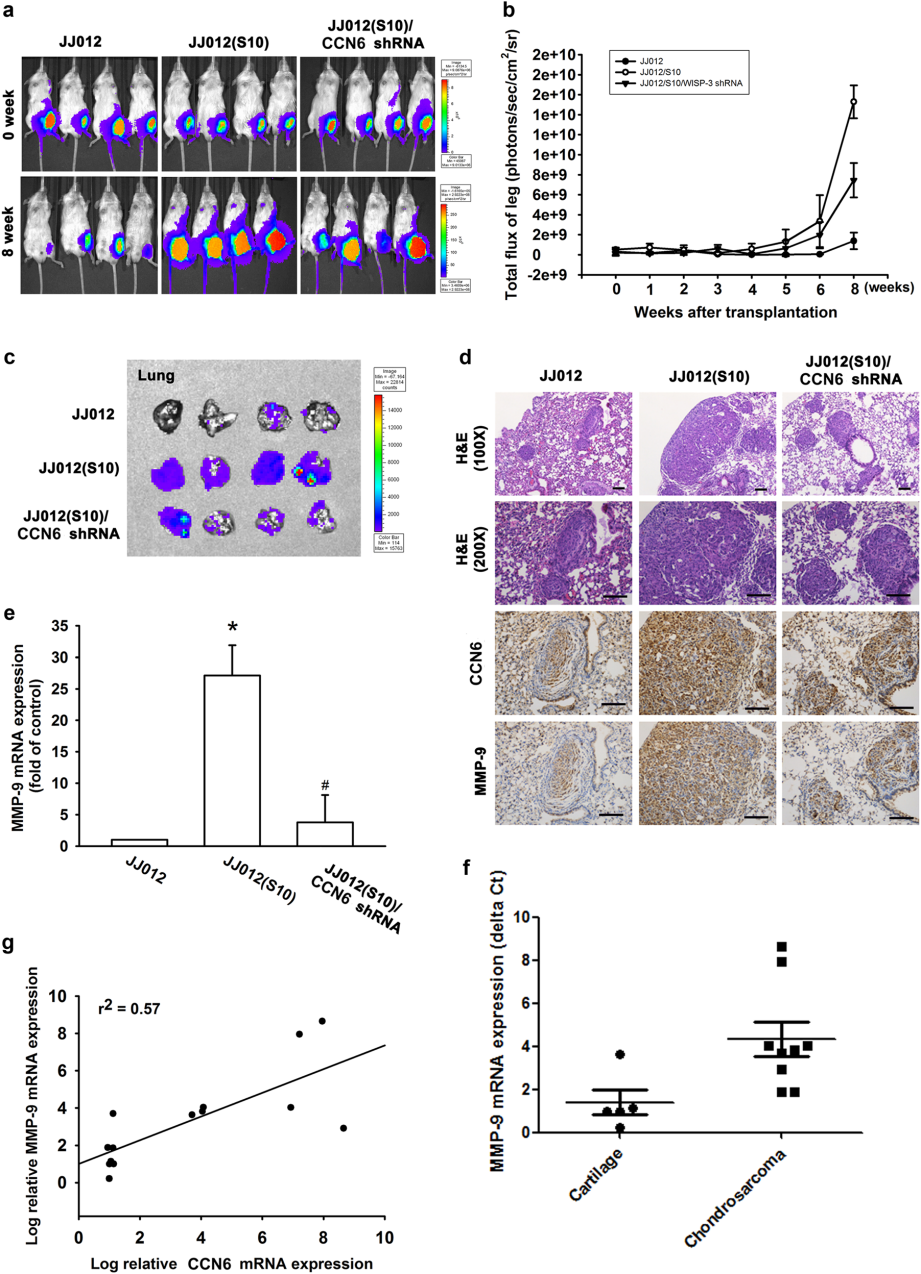
The correlation of CCN6, MMP-9 and metastasis in clinical and in vivo analysis. **a**–**c** The mice were injected with JJ012, JJ012(S10), or JJ012(S10)/CCN6 shRNA cells. Lung metastasis was monitored by bioluminescence imaging at the indicated time intervals, then quantified by photon images of the left leg region. After 8 weeks, these mice were humanely sacrificed and the lung tissue was excised, photographed and quantified. **d**, **e** CCN6 and MMP-9 protein and mRNA expression in these lung tumors were assessed by RT-qPCR and IHC analysis. Size bar = 100 μm. **f**, **g** CCN6 and MMP-9 mRNA expression in normal cartilage and chondrosarcoma tissue was examined by RT-qPCR. Quantitative results are expressed as the mean ± SEM. *p < 0.05 as compared with the control group; #*p* < 0.05 as compared with the CCN6-treated group

We developed an orthotopic mouse model of chondrosarcoma. JJ012 cells were transduced with luciferase and clonally selected for orthotopic implantation, then orthotopically implanted into the left leg tibia. Tumor size was calculated over time based on luminescence intensity (Fig. 7a, b). At 8 weeks, IVIS findings showed that knockdown CCN6 reduced tumor metastasis to the lung (Fig. 7c). IHC data revealed decreases in the expression levels of CCN6 and MMP-9 in the JJ012/CCN6 shRNA orthotopic model (Fig. 7d). Furthermore, we detected high human MMP-9 mRNA expression in lung tissue from the JJ012(S10) group of mice, and that knockdown human CCN6 lowered levels of human MMP-9 expression (Fig. 7e). RT-qPCR analysis of samples revealed significantly higher CCN6 and MMP-9 expression in chondrosarcoma patients compared with healthy controls (Fig. 7f)^23^. High levels of CCN6 expression correlated strongly with high MMP-9 expression (Fig. 7g). Our results indicate that endogenous CCN6 activates the PI3K/Akt/mTOR/NF-κB signaling pathway and augments MMP-9-dependent lung metastasis of chondrosarcoma in vivo.

## Discussion

Chondrosarcomas are a group of heterogeneous, malignant bone neoplasms that constitute around 26% of all bone cancers^24,25^. The metastatic potential of conventional chondrosarcomas correlates closely with the histologic tumor grade. Surgery is the main therapeutic option for chondrosarcoma; chemotherapy, and radiotherapy have very limited effectiveness^26^. Many low- and moderate-grade chondrosarcomas have a relatively indolent growth rate; approximately 15% of all metastatic disease-related deaths occur more than 5 years after initial diagnosis^27^. This characteristic offers an important opportunity for an effective adjuvant therapy to prevent metastatic disease in chondrosarcoma. We hypothesized that CCN6 would influence metastasis in chondrosarcoma cells. In this current study, stimulation of chondrosarcoma cell lines with CCN6 promoted cell motility and MMP-9 expression, which in turn activated the PI3K/Akt/mTOR/NF-κB signaling pathway. In addition, we observed a strong correlation between CCN6 and MMP-9 expression in human chondrosarcoma tissue and preclinical analyses. CCN6 may therefore be a novel target for chondrosarcoma metastasis.

The CCN protein family has been implicated in the enhancement of tumor proliferation, migration and metastasis^28^. For example, the knockdown of CCN6 expression suppressed gastric cancer cell proliferation and migration via the Wnt/β-catenin signaling pathway in an in vitro investigation, and high CCN6 expression has been linked to poor prognosis in patients with gastric cancer^29^. Interestingly, high CCN6 expression has the opposite effect in some tumors. In breast cancer cell lines, CCN6 overexpression inhibits cell growth and invasiveness, while CCN6 induces a epithelial to mesenchymal transition (EMT) and reduces tumor-initiating cells (TIC), which is mediated by the downregulation of Slug and inhibition of the Notch1 signaling pathway^30,31^. Thus, CCN6 molecular mechanisms appear to differ between different types of tumor cells.

Lethal outcomes associated with the vast majority of cancers is due to the dissemination of metastatic tumor cells and the outgrowth of secondary tumors at distant sites^11^. Metastasis is initiated through changes in cell–cell adhesion properties, rearrangement of the ECM environment, anoikis suppression, reorganization of the cytoskeleton, and tumor cell invasion into the peripheral tissue, resulting in cancer cell intravasation into blood or lymphatic vessels, enabling them to disseminate into secondary organs^18^. Tumor invasion and metastasis has to cross several physical barriers, such as the endothelial basement membrane. As described earlier, evidence indicates that MMPs play a role in the pathologic, metastasis-prone vasculature of growing tumors^11^. Our findings demonstrate CCN6-induced enhancement of MMP-9 expression and secretion in human chondrosarcoma cells; the loss of MMP-9 expression significantly suppressed CCN6-induced cell migration and invasion activity. Therefore, MMP-9 may act as the CCN6-responsive mediator, causing ECM degradation, which may lead to subsequent cancer migration and invasion activity.

Activation of the PI3K/Akt/mTOR signaling pathway is a central event in many types of cancer and represents a promising target for new treatment strategies. This pathway mediates multiple cellular functions, including cell survival, proliferation, migration, and autophagy^32^. Furthermore, the PI3K/Akt/mTOR signaling pathway is associated with tumor metastasis. For instance, CXCL6 secretion is increased by fibroblast-derived CXCL12/SDF-1α, which also enhances colonic cancer metastatic potential through the PI3K/Akt/mTOR pathway^33^. In this study, we demonstrated that PI3K, Akt, and mTOR inhibitors are capable of inhibiting CCN6-induced migration and invasion of cells, and reducing MMP-9 mRNA expression, which suggests that PI3K/Akt/mTOR activation has to occur for CCN6 to enhance MMP-9 expression and induce cell motility. Indeed, we observed that p85, Akt, and mTOR siRNAs reduced MMP-9 expression, and inhibited chondrosarcoma cells migration and invasion. When we incubated cells with CCN6, we observed an increase in PI3K, Akt, and mTOR phosphorylation. Pretreatment of cells with Ly294002, wortmannin, or an Akt inhibitor reduced CCN6-mediated Akt and mTOR phosphorylation. This indicates that the PI3K/Akt/mTOR signaling pathway is required for CCN6-induced MMP-9 expression and chondrosarcoma cell metastasis. In addition, we found that c-Src, MAPK but not AMPK are also involved in CCN6-induced chondrosarcoma cell migration^17^ (Supplementary Figure S2). Further investigations are needed to determine whether these or other signaling pathways mediate the effects of CCN6 on cell migration.

NF-κB controls the induction of MMP-9 transcription in human cancer cells^34,35^. Our data show that NF-κB activation plays an important part in CCN6-induced migration and MMP-9 expression in human chondrosarcoma cells and that blocking the NF-κB-dependent signaling pathway inhibits CCN6-induced MMP-9 expression and cancer metastasis. When we transfected chondrosarcoma cells with κB-luciferase, we found that CCN6 enhanced NF-κB activity. Treatment with Ly294002, wortmannin, an Akt inhibitor and rapamycin, or p85, Akt, and mTOR siRNAs reduced CCN6-induced increases in NF-κB promoter activity. This suggests that CCN6 increases MMP-9 expression and induces cell metastasis in human chondrosarcoma cells via the PI3K/Akt/mTOR/NF-κB signaling pathway.

Patients with metastatic chondrosarcoma have a very poor prognosis, so it is important to find a means of preventing metastasis^36^. Our results highlight new insights into CCN6 functions in the metastatic process. Upregulation of CCN6 expression promotes metastasis in chondrosarcoma. The mechanisms involved in CCN6-induced metastasis remain unclear, although our findings show that CCN6 acts via the PI3K/Akt/mTOR/NF-κB signaling pathway. This study emphasizes the importance of CCN6 in chondrosarcoma metastasis and suggests that CCN6 can be a useful target in the management of chondrosarcoma.

## Materials and methods

### Materials

We purchased p85α, Akt1, p-IKKα/β (Ser180/Ser181), p-IκBα, p65 (Ser536), IKKα/β, IκBα, p65, CCN6, and β-actin primary antibodies (Santa Cruz Biotechnology, CA, USA), and rabbit polyclonal antibodies specific for p-p85 (Y458), p-Akt (S473), p-mTOR (Ser2448), and mTOR (Cell Signaling Technology, Danvers, MA, USA). Recombinant human CCN6 was purchased from PerpoTech (Rocky Hill, NJ, USA) and CCN6 short hairpin (sh)RNA expression plasmids from RNAiCore (Taipei, Taiwan). The D-Luciferin potassium salt was purchased from Gold Biotechnology (St. Louis, MO, USA). Lipofectamine 2000 and TRIzol were purchased from Life Technologies (Carlsbad, CA, USA). Dharmacon Research (Lafayette, CO, USA) supplied ON-TARGETplus siRNAs. Gibco-BRL life technologies (Grand Island, NY, USA) supplied DMEM, α-MEM, fetal bovine serum (FBS), and all other cell culture reagents. The Matrigel was purchased from BD (Biosciences, Bedford, MA, USA) and Promega (Madison, WI,) supplied the pSV-β-Galactosidase Vector and luciferase assay kits. All other USA chemicals or inhibitors that we used were supplied by Sigma-Aldrich (St. Louis, MO, USA).

### Cell culture

The human chondrosarcoma cell line (JJ012) was kindly supplied by Dr. Sean P. Scully’s laboratory at the University of Miami School of Medicine (Miami, FL, USA). We selected the highly migratory JJ012(S10) cells in our laboratory^37^. The human chondrosarcoma cell line (SW1353) was obtained from the American Type Culture Collection. Cell culture conditions were recorded as previously described^37^.

### Migration and invasion assay

Cell migratory and invasion ability was examined with 24-Transwell culture plates (Costar, NY, USA; pore size, 8 μm), with some modifications^18^. For the invasion assay, filters were pre-coated with 30 μL Matrigel basement membrane matrix for 1 h. For the migration and invasion assays, we placed 1.5 × 10^4^ cells into the upper Transwell chamber and varying concentrations of CCN6 and inhibitors into the lower chamber. Cells were incubated for 18 h in a humidified incubator (at 37 °C, 5% carbon dioxide [CO_2_]), then fixed in 3.7% formaldehyde solution and stained with 0.05% crystal violet. We used a microscope to visually count the cells in the lower chamber.

### Quantitative real-time polymerase chain reaction (RT-qPCR)

We used a TRIzol kit (MDBio Inc., Taipei, Taiwan) to extract total RNA from chondrosarcoma cells. Next, we synthesized cDNA with an Invitrogen reverse transcription kit (Carlsbad, CA, USA)^38^. qPCR analysis was performed with the Taqman^®^ One-Step RT-PCR Master Mix (Applied Biosystems, CA, USA). We added 2 μL of cDNA template to each 25 μL reaction using sequence-specific primers and Taqman^®^ probes. GAPDH mRNA expression served as an internal control^39^. The sequences of the primers were as follows: MMP-9 forward 5’-CACTGTCCACCCCTCAGAGC-3′, and MMP-9 reverse 5’-GCCACTTGTCGGCGATAAGC-3’. GAPDH forward 5′-ACCACAGTCCATGCCATCAC-3′, and GAPDH reverse 5′-TCCACCACCCTGTTGCTGTA-3′.

### Western blotting

Human chondrosarcoma cells were plated in 6-well plates at a density of 2 × 10^5^ cells/well in culture medium. Cellular lysates were prepared as previously described^40,41^. Proteins were resolved on sodium dodecyl sulfate-polyacrylamide gel electrophoresis (SDS-PAGE) then transferred to polyvinylidene fluoride (PVDF) membrane filters. Blot membranes were blocked with Tris-Buffered Saline-Tween 20 (TBST) containing 4% non-fat milk for 1 h at room temperature, then incubated with primary antibodies for 1 h and washed 3 times with TBST for 5 min each, and incubated for 1 h at room temperature with HRP-conjugated anti-rabbit or anti-mouse secondary antibodies. We visualized the blot membranes with a Fujifilm LAS-3000 imaging system (Fujifilm, Tokyo, Japan).

### siRNA transient transfection and reporter gene assay

Dharmacon Research (Lafayette, CO, USA) supplied ON-TARGETplus siRNAs for p85, Akt, mTOR, p65, MMP-9 and control. We transiently infected siRNAs (100 nM) with DharmaFECT1 transfection reagent.

Chondrosarcoma cells were seeded on 12-well plates at a density of 1 × 10^5^ cells/well, grown to 80% confluence and transfected the next day. We used Lipofectamine 2000 to transfect NF-κB luciferase plasmid into cells, which were then incubated with the indicated agents. Cell extracts were analyzed for luciferase and β-galactosidase activities.

### Zymography

Conditioned medium was collected and electrophoresed through a polyacrylamide gel containing 0.25% gelatin. The gel was washed twice for 15 min at room temperature with 2.5% Triton X-100. Subsequently, the gel was incubated at 37 °C overnight in a buffer containing 150 mM NaCl, 50 mM Tris–HCl, and 10 mM CaCl_2_, pH 7.5. The gel was stained with 0.2% Coomassie blue and photographed on a light box. Proteolysis was detected as a white zone in a blue field.

### Immunofluorescence

Cells were cultured in 12-well dish at a density of 3 × 10^3^ cells/ coverslips. Cells were pretreated with different inhibitors for 30 min then stimulated with CCN6 for 60 min. The cells were fixed with 4% paraformaldehyde at room temperature. Thirty minutes later, 0.5% Triton X-100 was added to the cells. The cells were then incubated with rabbit anti-p65 (1:100) and FITC-conjugated goat anti-rabbit secondary antibody (1:100; Leinco Technologies Inc) for 1 h. Finally, cells stained with DAPI for 5 min were washed, mounted, and examined with a Zeiss fluorescence microscope.

### In vivo tumor xenograft study

All animal experiments satisfied the protocols specified by China Medical University’s Institutional Animal Care and Use Committee (IACUC Approval No. 104-154-N). Four-week-old male BALB/c nude mice were purchased from Taipei’s National Laboratory Animal Center and randomly assigned to one of the following orthotopic implant groups: JJ012, JJ012(S10), or JJ012(S10)/CCN6 shRNA. Under isoflurane anesthesia (1.5–2.5%), JJ012, JJ012(S10), or JJ012(S10)/CCN6 shRNA cells (5 × 10^6^, resuspended in 50 μL of medium containing 50% serum-free DMEN/α-MEM and 50% Matrigel) were orthotopically implanted into the left leg tibia of each mouse. Tumor growth in the tibiae was monitored for 8 weeks by bioluminescence imaging using a Xenogen IVIS imaging system 200 (PerkinElmer, MA, USA). Prior to imaging, the mice were anesthetized with isoflurane (1.5–2.5%) and then intraperitoneally injected with D-Luciferin potassium salt 150 mg/kg. Images were analyzed using Living Image 4.0 software (Caliper, Alameda, CA). At 8 weeks, the mice were euthanized by CO_2_ inhalation. The lungs were removed and fixed in 10% formalin for further analysis.

### Immunohistochemical (IHC) staining

CCN6 or MMP-9 protein expression was determined by IHC staining of tissue slides^37^. Tissue sections were deparaffinized with xylene and rehydrated with ethanol. Endogenous peroxidase activity was blocked with 3% hydrogen peroxide. Using the antigen retrieval buffer system for IHC staining, we incubated the slides with primary antibodies against CCN6 or MMP-9. The antibody binding signal was detected by the NovoLink Polymer Detection System (Leica Microsystems, Heidelberg, Germany) and visualized using 3-3’-diaminobenzidine. The sections were counterstained with hematoxylin.

### Patients with chondrosarcoma and specimen preparations

The study protocol was approved by China Medical University Hospital’s Institutional Review Board (CMUH 104-REC2-055, CMUH103-REC2-023). Written informed consent was obtained from all study participants prior to study commencement. Tumor tissue specimens were collected from patients with chondrosarcoma undergoing surgical resection procedures and human cartilage tissues were obtained from otherwise healthy OA patients undergoing knee replacement surgery; all procedures were performed in China Medical University Hospital. Total RNA was extracted from tissue using a TRIzol kit (MDBio, Taipei, Taiwan), and all unused human tissue was stored at –80 °C.

### Statistical analysis

All data are presented as the mean ± standard error of the mean (SEM) of at least three independent experiments. Student’s *t*-test determined statistical differences between samples and the Bonferroni post hoc procedure was performed for a one-way analysis of variance (ANOVA) of statistical comparisons between more than two samples. A *p*-value of <0.05 was considered statistically significant.

## Electronic supplementary material

Supplementary data
