# Supplementary material for: CCN6-mediated MMP-9 activation enhances metastatic potential of human chondrosarcoma
Source: Cell Death Dis. 2018 Sep 20;9(10):955. doi: 10.1038/s41419-018-1008-9 (PMC6147788; doi:10.1038/s41419-018-1008-9)
Supplement: Supplementary file 1 — Supplementary data [file 41419_2018_1008_MOESM1_ESM.doc]

**Supplementary data**

**Fig. S1.**

**
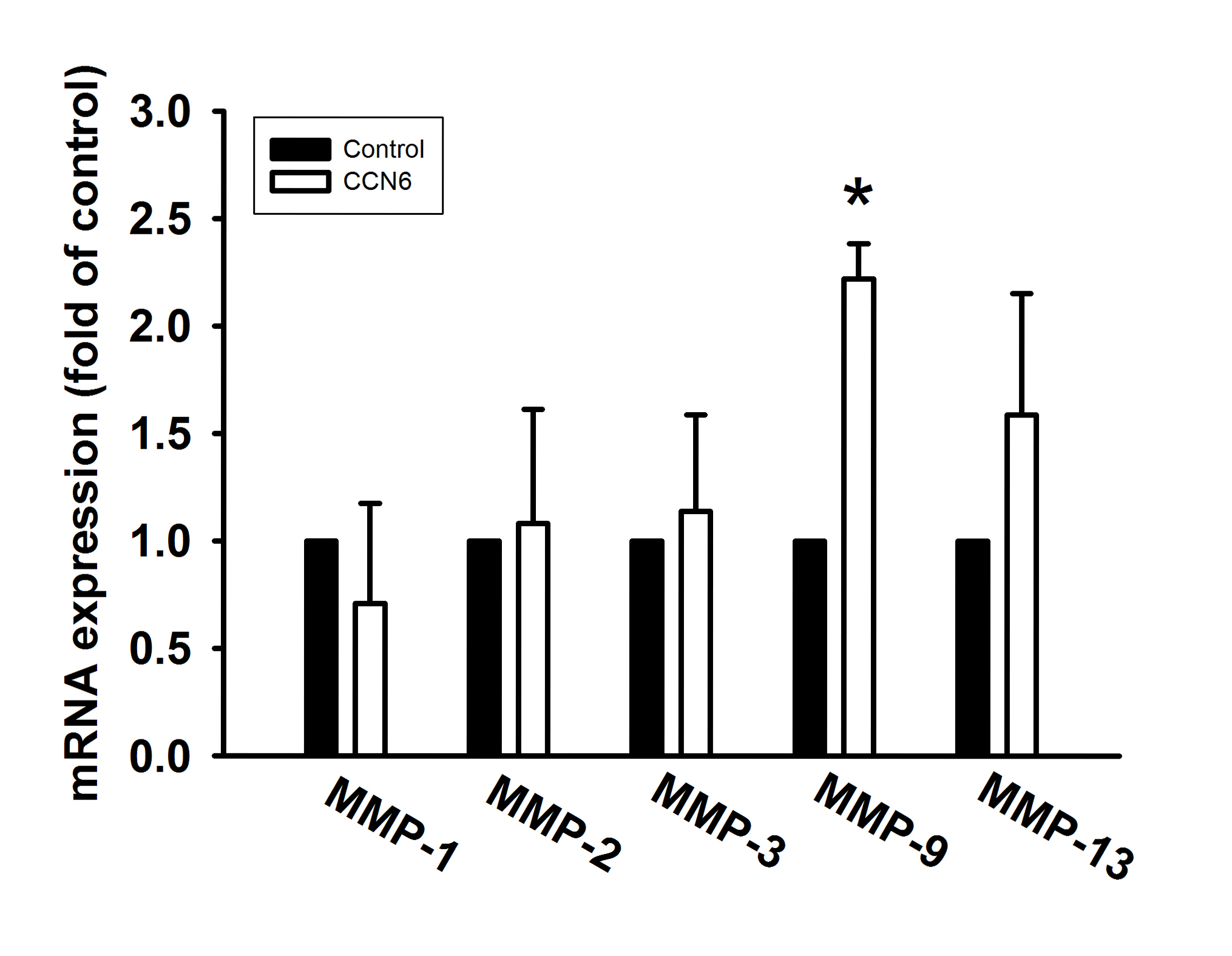
**

**Figure S1. CCN6 induced MMP-9 mRNA expression of chondrosarcoma cells.** JJ012 cells were incubated with CCN6 (50 ng/ml) for 24 h, the mRNA level of MMP-1, -2, -3, -9, and -13 was determined using qPCR. Quantitative results are expressed as the mean ± SEM. **p* < 0.05 as compared with the control group.

**Fig. S2.**


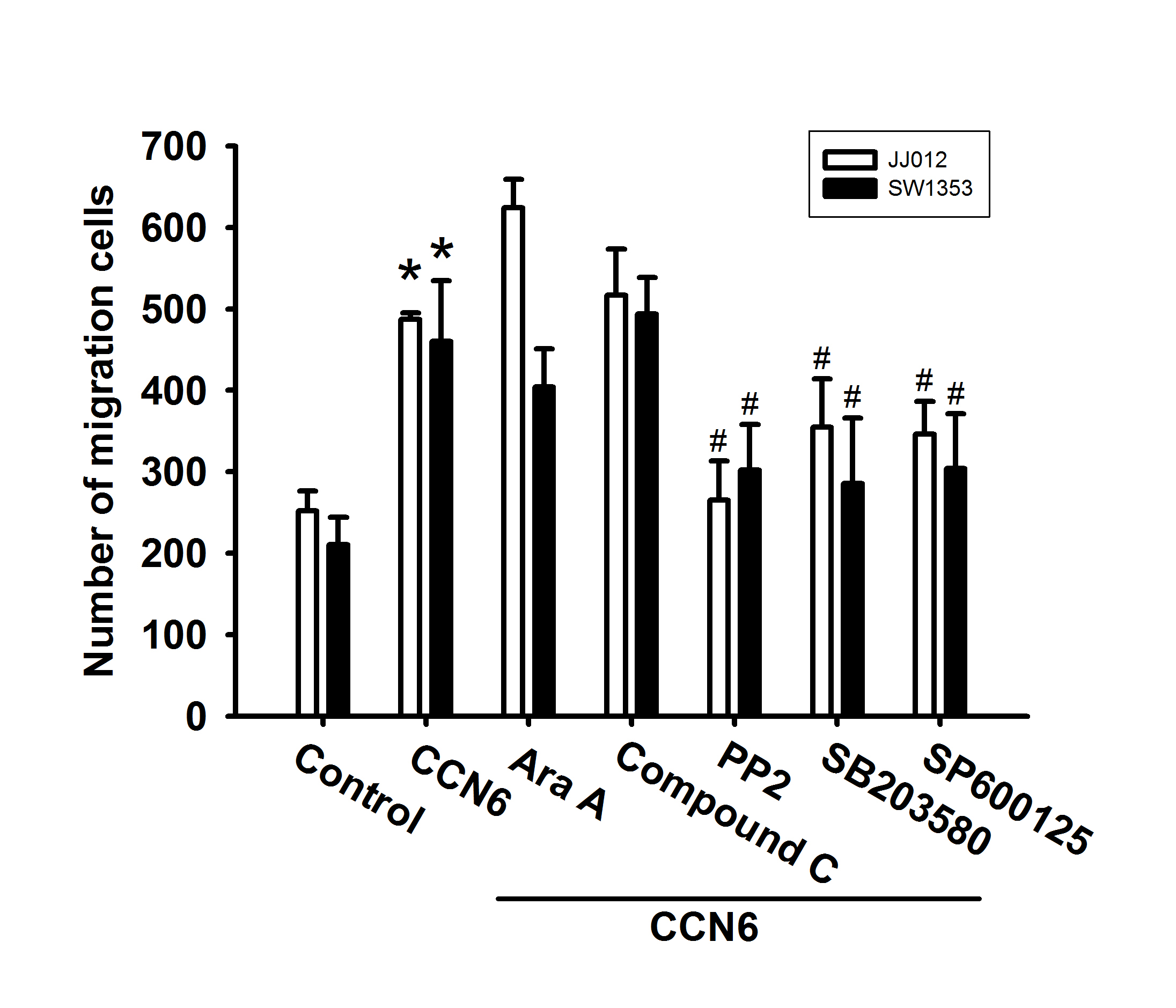


**Figure S2. The c-Src, MAPK signaling proteins are involved in CCN6-induced chondrosarcoma cells migration.** Cells were pretreated for 30 min with Ara A (10 μM), Compound C (20 μM), PP2 (3 μM), SB203580 (10 μM), and SP600125 (10 μM) for 16 h, then stimulated with CCN6. The cells migration was examined by Transwell assay. Quantitative results are expressed as the mean ± SEM. **p* < 0.05 as compared with the control group.
